# Do emotional difficulties and peer problems occur together from childhood to adolescence? The case of children with a history of developmental language disorder (DLD)

**DOI:** 10.1007/s00787-018-1261-6

**Published:** 2018-12-05

**Authors:** Gina Conti-Ramsden, Pearl Mok, Kevin Durkin, Andrew Pickles, Umar Toseeb, Nicola Botting

**Affiliations:** 1 Human Communication, Development and Hearing (HCDH), School of Health Sciences, The University of Manchester and Manchester Academic Health Science Centre, Ellen Wilkinson Building, Oxford Road, Manchester, M13 9PL UK; 2 University of Strathclyde, Glasgow, UK; 3 King’s College London, London, UK; 4 University of York, York, UK; 5 City, University of London, London, UK

**Keywords:** Emotional health, Peer problems, Developmental language disorder (DLD), Longitudinal studies, Developmental psychopathology, Child development

## Abstract

Children and adolescents with developmental language disorder (DLD) are, overall, vulnerable to difficulties in emotional adjustment and in peer relations. However, previous research has shown that different subgroups follow different trajectories in respect to the quality of peer relations. Less is known about the trajectories of emotional development. We consider here the possibility that development in these two domains is interrelated: that is, the trajectories of emotional and peer problems will proceed in parallel. We conducted longitudinal joint trajectories analyses of emotional and peer relations in a sample of young people identified as having DLD at the age of 7 years and seen at intervals up to 16 years. Potential influences on joint trajectory group membership were examined. Findings revealed five distinct joint trajectories. Emotional and peer difficulties do occur together from childhood to adolescence for just over half of the sample, but not all. The variables most clearly associated with group membership were pragmatic language ability, prosociality and parental mental health. This is the first study to examine joint longitudinal trajectories of emotional and peer difficulties in individuals with DLD. We demonstrate that development in individuals with DLD is heterogeneous and identify three key variables associated with personal and social adjustment from childhood to adolescence. Theoretical and clinical implications of these findings are discussed.

**Electronic supplementary material:**

The online version of this article (10.1007/s00787-018-1261-6) contains supplementary material, which is available to authorized users.

## Introduction

Children with developmental language disorder (DLD) have no hearing disabilities and show no evidence that their language difficulties associated with a known biomedical aetiology (such as cerebral palsy) [1]. Some 7–10% of children in the UK enter school with impaired language abilities [2].

Notwithstanding the absence of neurological abnormalities and cognitive deficits, children and adolescents with histories of DLD do show a heightened risk of various other developmental difficulties. For example, as a group, they tend to manifest higher levels of conduct disorder and hyperactivity than do typically developing peers [3, 4]. They are prone to greater difficulties in peer relations and friendships [5–7]. They also have higher levels of mental health difficulties, such as anxiety, fearfulness, depressive symptoms and panic [8, 9].

One area of particular vulnerability for children and adolescents with DLD is emotional regulation. Compared to typical peers, these young people are almost twice as likely to show clinical levels of emotional difficulties [5, 10]. A meta-analysis of existing evidence suggests that, on average, children with DLD are above the 70th percentile on severity of emotional difficulties [11]. With the exception of very early childhood, between the ages of 4 and 7 years [12], longitudinal studies have found higher levels of emotional difficulties in DLD not only across childhood but into young adulthood [3, 11]. The accumulating evidence indicates a clinically important connection between DLD and the development of emotional difficulties.

The studies available to date are informative of the overall trajectory of emotional difficulties in DLD. Comparisons of results across studies indicate that trajectory of emotional difficulties in DLD appear stable across time, with a modest increase in difficulties with age. Such a trajectory of emotional difficulties is consistent with those found in general population studies [13, 14]. It is important to note, however, that some investigations that have examined childhood baseline levels of emotional difficulties and later emotional outcomes in DLD have not found stability.

Some investigators have reported longitudinal increase in symptomatology [8], whilst others have found amelioration/resolution of difficulties [15] and still others have reported curvilinear patterns, i.e., decrease followed by increase [16]. Although such inconsistencies are likely to reflect, at least in part, differences in the samples studied and methodological differences with respect to participants’ ages and measures used, they may also indicate individual differences. There may be groups of children with DLD that experience different developmental trajectories of emotional difficulties. DLD is known to be heterogeneous; different children manifest different areas and/or combinations of language difficulties in respect of expression, comprehension, and pragmatic performance [1, 17].

We also know that there is variability in the ways in which DLD is associated with developmental difficulties in other domains of functioning, such as behaviour or social interactions [3]. In the social domain, Mok et al. [7] have documented clear differences in the development of difficulties with peer interactions. One group of children with DLD in that study experienced problems with peers from childhood through adolescence (persistent). Another group had peer difficulties in childhood that appeared to resolve in adolescence (childhood limited). Another group experienced an increase in peer problems from early adolescence (adolescent onset). Other children experienced relatively modest peer difficulties throughout the same period (low/no problems). In the present study, we ask whether similar trajectories are identifiable in respect of emotional difficulties in children with DLD and whether the trajectories followed in respect of emotional difficulties are aligned with those identified in respect of peer relations: that is, do problems in one of these areas invariably signify that problems are likely in the other?

There is some evidence to indicate that emotional and peer problems are associated in childhood and adolescence in general [18, 19], and this has been reported in DLD populations in particular [7]. Mok et al. [7] found that, with respect to peer problems, children in the childhood-onset persistent problems group and those with adolescent-onset problems showed higher levels of emotional symptoms than those with low/no problems. On this evidence, then, it appears that these difficulties are interwoven. What is less clear is how they are interrelated across development. For example, a relatively straightforward expectation could be that difficulties in each domain develop in parallel, due either to one type of problem precipitating the other (e.g., children with emotional difficulties are less able to form and maintain successful peer relations), or because the variables are linked bi-directionally (i.e., each problem type exacerbating the other over time: emotional difficulties impact on peer relations and vice versa), or they share common etiological factors which affect growth of both emotional and peer problems. A more complex possibility is that different children show different patterns of joint trajectories. That is, some may manifest parallel developments across peer relations and emotional regulation, while others may show divergent trajectories. Relatively little research has been conducted into co-occurring developmental trajectories, but the issue is crucial to advancing our understanding of developmental relations and to informing diagnosis and clinical interventions [18, 19]. Hence, a principal purpose of this investigation was to determine whether these two areas of problematic development occur together over time.

Another aim of this study was to examine potential factors associated with developmental trajectories of emotional and peer problems from childhood to adolescence.

One possibility involves the consequences of facing adolescence with the burden of persisting language difficulties. We examined expressive, receptive and pragmatic language skills and hypothesized that severity of language disorder would be associated with increased difficulties in adolescence. This is because research with children with DLD suggests that language skills, and in particular pragmatic skills, are associated with how well children comprehend emotions and emotional descriptions, how well they self-regulate their own emotions [20, 21] and whether they engage in successful peer relations and friendships [3, 7]. We also anticipated that social abilities are likely to play a role in the progress of emotional difficulties. Problems with peer interactions have been shown to be associated with increasing levels of emotional difficulties [7, 22] whilst prosociality is positively associated with emotional adjustment [23, 24]. Hence, we expected that lower prosociality in later childhood would be associated with less favourable joint trajectories, namely persistent problems in emotional and peer relations throughout childhood into adolescence and adolescent-onset problems, i.e., increasing problems in these domains during adolescence.

Other factors are known to bear on vulnerability to emotional difficulties which may also bear on social adjustment. These include gender [14, 25] and parental history of mental health difficulties [26]. Population studies have revealed that an increase in emotional difficulties in adolescence is more pronounced in girls [13, 14]. On this basis, we predicted that there would be a larger proportion of girls with DLD with adolescent-onset emotional difficulties. Parental mood and anxiety disorders are known to be associated with increasing levels of emotional, social and behavioural difficulties in their offspring [22, 27, 28]. Hence, we expected an association between parental mental health difficulties and increasing symptomatology, such that higher indications of parental mental health difficulties would be associated with the less favourable joint trajectories of emotional and peer problems.

## Method

### Participants

The participants in this study have a history of DLD and were originally part of a wider longitudinal study [29, 30] the Manchester Language Study (MLS). The initial cohort of 242 children (6;6–7;9 years) was a random sample of 50% of all 7-year-olds attending 118 language units across England. Language units (usually attached to mainstream schools) are specialised classes for children who have been identified with primary speech and language difficulties. Children were excluded from the study if teachers reported frank neurological difficulties, hearing impairment, a diagnosis of autism or a general learning disability. Thus, children with low nonverbal abilities were most likely excluded from attending language units.

Participants were contacted again at ages 8 (*N *= 232), 11 (*N* = 200), 14 (*N* = 113), and 16 (*N* = 139). Ethical approval was obtained from The University of Manchester and written informed consent was gained from all participants at each stage. The attrition observed was partly due to funding constraints at follow-up stages of the study. Participants for the follow-up stages of the study were retained mainly on the basis of traceability and geographical accessibility. There were no significant differences in receptive language, expressive language, performance IQ (PIQ), household income, emotional difficulties, or peer problems at age 7 between those who participated at age 16 and those who did not, *p* > .1.

Measures of teacher-reported emotional difficulties were available at ages 7, 8, 11 and 16. Only individuals who had these measures for at least three of the four time points, and in addition had measures of peer problems, were included: a total of 168 children (24% girls). The participants’ psycholinguistics profiles at 7, 11, and 16 years of age are presented in Table 1. Data revealed the average standard scores for receptive language at all three ages and for expressive language at age 7 were around 1 SD below the population mean, whilst average expressive language scores at ages 11 and 16 were more than 1.5 SD below. Mean PIQ scores fell between ages 7 and 11 [31, 32]. At age 7, PIQ was above the population mean. By age 11, on average, PIQ was lower (approximately − 1 SD) and remained at a similar level at age 16. No children from the original study were excluded at later stages, since there is evidence suggesting that children with low PIQ and language skills perform much like children with DLD who have PIQ within the normal range [1, 33]. In the original MLS sample, 53% of the participants came from households earning less than the average family wage for that year and 47% came from households earning more than this threshold.

**Table 1:** Mean (SD) of language and PIQ scores of children at ages 7, 8, 11 and 16

	Age 7	Age 8	Age 11	Age 16
Receptive language standard scores^a^	83.6 (11.3)	85.5 (12.4)	86.6 (15.6)	83.1 (16.5)
Expressive language standard scores^b^	83.2 (10.0)	83.8 (11.3)	73.7 (11.7)	73.1 (10.6)
PIQ standard scores^c^	105.5 (15.0)	108.2 (15.7)	85.8 (23.6)	83.7 (18.9)

^a^Receptive language measures at ages 7, 8 and 11: test for reception of grammar [39]; age 16—word classes subset of the clinical evaluation of language fundamentals [41]

^b^Expressive language measures: ages 7 and 8—Bus Story Test [40]; age 11 and 16—Recalling Sentences subtest of the Clinical Evaluation of Language Fundamentals—Revised [41]

^c^PIQ measures: age 7 and 8—Raven’s coloured progressive matrices [37]; age 11—Block Design and Picture Completion of the Wechsler Intelligence Scale for Children—third edition [38] and at age 16 the full form of the Wechsler Intelligence Scale for Children—third edition [38]

Although all the children had been identified as having significant language problems on entry to the language units, their language profiles were heterogeneous and susceptible to changes over the course of the longitudinal study. Participants thus had a history of DLD, however, for simplicity participants will be referred to as children with DLD. In addition, it is known that DLD is a heterogeneous condition, thus it is not surprising that historically, different diagnostic terminology has been used to describe this group including the terms language impairment (LI), developmental language disorder (DLD), and specific language impairment (SLI). Longitudinal studies in this area, including the Manchester Language Study, have also reflected in their publications the historical changes in terminology used with this population [29]. In line with current recommendations, following a Delphi consensus study focusing on characteristics, diagnosis and terminology in this area [1], this paper will use the term DLD throughout.

### Instruments and measures used

#### Measures of emotional difficulties

The Rutter Children’s Behaviour Questionnaire [34], completed by the children’s teachers at ages 7, 8 and 11, was used to assess emotional difficulties. The questionnaire consists of 26 statements and the child’s teacher is asked to score each item as ‘doesn’t apply’(0), ‘applies somewhat’(1) or ‘certainly applies’(2). Scores of five items (the four items constituting the Rutter neurotic subscale: ‘Often worried, worries about many things’, ‘Often appears miserable, unhappy, tearful or distressed’, ‘Tends to be fearful or afraid of new things or new situations’, and ‘Has had tears on arrival at school or has refused to come into the building this year’, as well as of the item ‘Often complains of pains or aches’) were summed to give a measure of emotional difficulties at each of the three ages. Using this method, scores derived ranged from 0 to 10, with higher scores indicating increasing emotional difficulties.

Emotional difficulties at ages 11 and 16 were assessed using the emotional difficulties subscale of the teacher-reported version of the Strengths and Difficulties Questionnaire (SDQ) [35] which was based on the Rutter questionnaire and retained several of the same items. Thus, we had two measures of emotional difficulties at age 11 (Rutter and SDQ). The SDQ is a 25-item behavioural questionnaire. The 25 items are divided between 5 subscales of 5 items each, with each item being coded as ‘not true’, ‘somewhat true’ or ‘certainly true’. The emotional difficulties subscale consists of the five items: ‘Often complains of headaches, stomach aches or sickness’, ‘Many worries, often seems worried’, ‘Often unhappy, downhearted or tearful’, ‘Nervous or clingy in new situations, easily loses confidence’, and ‘Many fears, easily scared’. Total scores on the subscale range from 0 to 10, with higher scores indicating increasing emotional difficulties. Emotional difficulties scores can also be classified as ‘normal’ (0–4), ‘borderline’ (5) and ‘abnormal’ (6–10).

Scores derived from the Rutter questionnaire and from the SDQ have been found to be highly correlated and to have equivalent predictive validity [36]. In addition, a review of 48 studies on the reliability and validity of the SDQ found that both the parent and teacher versions have satisfactory internal consistency, test–retest reliability, inter-rater agreement, and good validity [36]. It concluded that the psychometric properties of the SDQ are strong, particularly for the teacher version.

#### Measures of problems in peer relations

The peer problems data reported by Mok et al. [7], were used for the comparative purposes of this study. Mok et al. used teacher-reported Rutter Children’s Behaviour Questionnaire and the teacher-reported version of the Strengths and Difficulties Questionnaire to measure peer problems. Unlike the SDQ, there is no peer problem subscale in the Rutter questionnaire. To derive a peer problem score using the latter, ordinal logistic regression analysis was conducted to investigate which Rutter items can significantly predict the SDQ peer problem subscales at age 11, i.e., the time point when both tests were administered. Three Rutter questionnaire items were significant predictors: ‘Not much liked by other children’ [Wald test: Chi square (2) = 55.5, *p* < .001], ‘Tends to do things on his/her own—rather solitary’ [Chi square (2) = 51.9, *p* < .001], ‘Bullies other children’ [Chi square (2) = 7.13, *p* = .028]. To derive a peer problem score for ages 7 and 8, ratings for the three items at each age were summed. Using this method, scores derived could range between 0 and 6, with higher scores indicating poorer peer relations. Similarly, a Rutter-based peer problem score was also derived for age 11, giving two measures of peer relations at that age, which were highly correlated, *r *= 0.82, *p* < .001. The peer problem subscale of the Strengths and Difficulties Questionnaire [35] consists of the five items: ‘Rather solitary, tends to play alone’, ‘Has at least one good friend’, ‘Generally liked by other children’, ‘Picked on or bullied by other children’ and ‘Gets on better with adults than with other children’. Total scores on the peer problem subscale range from 0 to 10; positive items are reverse-scored and higher scores indicate greater difficulties with peer relations. Peer problem scores can also be classified as ‘normal’ (0–3), ‘borderline’ (4) and ‘abnormal’ (5–10).

#### Performance IQ (PIQ)

Raven’s Coloured Progressive Matrices was used to assess participants’ PIQ at ages 7 and 8 [37]. At age 11, Block Design and Picture Completion of the Wechsler Intelligence Scale for Children—Third Edition (WISC-III UK) [38] was administered. At age 16, PIQ was assessed using the full form of the same test used at 11 [38].

#### Receptive and expressive language

At ages 7, 8 and 11, receptive language was assessed using the Test for Reception of Grammar [39]. Expressive language at ages 7 and 8 was assessed using the Bus Story Test [40] and at age 11, it was measured by the Recalling Sentences subtest of the Clinical Evaluation of Language Fundamentals-Revised (CELF-R) [41]. At age 16, language skills were assessed using The Word Classes subtest (receptive measure) and the Recalling Sentences subtest (expressive measure) of the CELF-R. It is important to note that although recalling sentences measures were used in this study to represent expressive language skills, this test also taps into reception, working memory and other language domains.

#### Pragmatic language

Pragmatic language skills were assessed at age 11 using the original version of the Children’s Communication Checklist [42]. The checklist consists of 70 items, grouped into 9 scales. Five of the subscales are concerned with pragmatic aspects of communication (inappropriate initiation, coherence, stereotyped conversation, context, and rapport). Each scale consists of a number of behavioural items which teachers or speech-language pathologists complete about the child based on their knowledge about the individual after at least 3 months. Professionals are asked to rate as ‘does not apply’, ‘applies somewhat’, or ‘definitely applies’. A composite pragmatic impairment scale formed from the five subscales had inter-rater reliability and internal consistency of around 0.80. A score of 132 or below is used as evidence for pragmatic language impairments. The mean score for the participants at age 11 was 140.8 (SD = 12.4). Of the 141 children included in this analysis, 32 (23%) met the criteria for pragmatic language impairments according to the CCC.

#### Prosociality

Prosocial behaviour subscale scores were obtained from the teacher-reported version of the SDQ questionnaire at age 11 [35]. Each of the SDQ subscales has five items and scores range from 0 to 10. For the prosocial subscale, the higher the rating, the more prosocial the individual. Examples of items constituting the prosocial subscale include: ‘Considerate of other people’s feelings’, ‘Kind to younger children’ and ‘Usually shares with others’.

#### Parental mental health

Parental mental health measures were obtained when the children with DLD were 14 years. The Family History Interview (FHI) [43] was used to document parental mental health. The FHI is an investigator-based interview schedule that elicits information on social and other psychiatric symptomatology in family members. The FHI was administered to both parents. Six questions were selected from the interview for the purposes of the present analyses. These questions covered the presence of depression, anxious worrying and generalised anxiety disorder in both childhood and adulthood. Each question is structured in terms of a definition that specifies the focus and scope of the item, together with criteria to set the severity threshold used for coding. In each case, there are one or more mandatory probes to provide a comparable orienting introduction to the item for the informant. The interviewer’s task is to obtain a description of behaviour that is sufficiently precise for a decision to be made on whether or not the specified criteria for the item are met. The interviewers were trained by the authors of the FHI over the course of 1 week before collecting any data on the field. For the purposes of this study, positive coding of these descriptions for any of the above emotional health disorders were combined, resulting in a single score on a scale of 0–12 (0 = neither parent had childhood or adulthood emotional health disorder; 12 = both parents had all three emotional health disorders in both childhood and adulthood). In addition, the percentage of families where both parents were affected either in childhood or in adulthood was also recorded. Importantly, there were no significant differences in the pattern of missing FHI data between the trajectory groups identified in this study.

### Statistical analyses

To examine whether emotional difficulties occur closely to peer relation problems, we undertook a joint trajectory analysis (a multivariate latent class growth model) to distinguish groups of children who shared common underlying levels and trajectories of emotional difficulties and problems in peer relations. All statistical analyses were conducted within Stata/SE 12.0 [44]. The ‘gllamm’ (generalized linear latent and mixed models; www.gllamm.org) [45] procedure command was used to model the changes in emotional difficulties and peer relations scores across time, identifying latent classes comprising children with similar patterns of development [46]. The scores were modelled using a mixed Poisson regression with the mean score being allowed to vary on the basis of the intercept (relating to the overall level/severity of the emotional difficulties), linear trends (allowing for differences in linear trajectory), and quadratic trends (allowing for differences in curvilinear trajectory). The models were then run with an increasing number of latent classes (referred to as “groups” henceforth) with each having a different intercept and linear trend. In addition, to allow for the use of different questionnaire measures earlier and later in the study (Rutter and SDQ), the models included a dummy variable for measuring in the fixed (mean) part of the model. With a log-link function, this acts to rescale the shared fixed and random parts of the linear predictor that define the trajectory of each class to the response range of each questionnaire. The model is thus a discrete class factor growth curve model for an overdispersed count. The joint modelling approach that we adopted was different to the usual approach to joint trajectory modelling, which is essentially one of correlated univariate models (i.e., one for emotional and one for peer problems), whereas we present trajectories through the bivariate space. Our approach is parametrically more efficient, treats the two problems as being intimately linked aspects of a potential common process, and was the parametrization used in our originating bivariate trajectories work [47].

For further analyses, we used both statistical goodness-of-fit criteria and interpretability, the latter taking into account the size of the groups and whether they captured forms of heterogeneity of clinical interest. The Akaike information criterion (AIC) and Bayesian information criterion (BIC), which penalizes more complex models, were used to assess the model fit. The most parsimonious model was the one with the lowest criterion value [48]. The chosen model was then used to calculate for each participant the empirical Bayes estimates for the posterior probability of belonging to each group, and each participant was assigned to the group with the highest posterior probability. All participants with data from both peer and emotional scores and three out of four time points were included (*n* = 168).

Sample attrition is a common problem in longitudinal studies, and the MLS is no exception. Attrition not only reduces the available sample size and thus statistical power, but where the attrition is selective can also introduce bias. The latent class growth models were fitted using full maximum likelihood to make use of all participants, both those with complete and incomplete data. There is, nonetheless, scope for bias in the simple overall sample means for measures at particular ages, however, conditioning on group—for example, examining the means by group—will account for much of this bias and weighting by group prevalence provides attrition-corrected estimates.

This investigation thus focuses on examining simultaneously two areas of functioning, namely emotional difficulties and peer relation problems. Examination of the developmental trajectories of a specific area of functioning has been published for peer relations problem [7]. Data on developmental trajectories of emotional difficulties specifically have not been published for these ages, thus we include these in the Supplementary Materials Appendix (Tables A1 and A2; Figure A1).

## Results

### Joint trajectory analysis: do developmental trajectories of emotional difficulties run in parallel to trajectories of problems in peer relations?

**Fig. 1:**
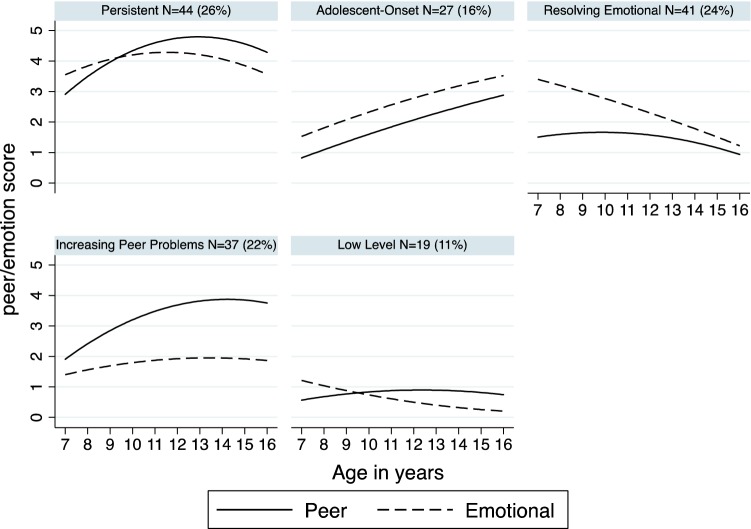
Predicted trajectories of joint peer–emotional difficulties

Table 2 presents the means and standard deviations for emotional difficulties and peer relations problems from childhood to adolescence. Table 3 provides the model statistics for the joint trajectory models run. We chose the five-class model as a parsimonious representation of the diversity of patterns of development of emotional difficulties and peer problems, and one where children were assigned with considerable confidence to their most likely trajectory class. Figure 1 presents the five groups of children with distinctive trajectories of emotional difficulties and peer problems. The patterns observed revealed that in approximately half the sample, the trajectories of emotional difficulties and peer relations problems do run in parallel from childhood to adolescence. Specifically, 26% of the total sample fell into the childhood-onset, persistent group in both domains (referred to as the persistent group), 16% fell into the adolescent-onset group in both domains (adolescent-onset group), and 11% showed consistently low scores in both domains (low levels group). For the other half of the sample, this was not the case. For one group (24% of the total sample), emotional problems were evident without accompanying peer problems, and these emotional difficulties were limited to childhood (resolving emotional group). For a further 22% of the total sample, peer problems increased from childhood and became more evident in adolescence, without accompanying emotional difficulties (increasing peer problems group). Thus, these two trajectory groups showed discrepancies in the development of emotional difficulties and peer problems.

**Table 2:** Mean (SD) emotional and peer problem scores

	Age 7	Age 8	Age 11	Age 16
Rutter emotional problems	1.79 (1.78)	2.17 (1.71)	2.22 (1.81)	–
SDQ emotional problems	–	–	2.63 (2.12)	2.43 (2.32)
Rutter peer problems	0.90 (1.06)	1.10 (1.20)	1.40 (1.30)	–
SDQ peer problems	–	–	2.72 (2.25)	2.94 (2.41)

**Table 3:** Model fit statistics and the number and percentages of children assigned to each group (joint trajectories)

Number of groups	AIC	Sample size corrected AIC	BIC	Average assignment probability	Number (%) of individuals					
					1	2	3	4	5	6
2	5336.44	5338.80	5377.05	0.93	98 (58%)	70 (42%)				
3	5244.40	5248.99	5300.63	0.89	63 (38%)	62 (37%)	43 (26%)			
4	5188.73	5196.40	5260.58	0.89	45 (27%)	61 (36%)	44 (26%)	18 (11%)		
**5**	**5160.68**	**5172.36**	**5248.15**	**0.86**	**44 (26%)**	**27 (16%)**	**41 (24%)**	**37(22%)**	**19 (11%)**	
6	5156.02	5172.76	5259.11	0.84	45 (27%)	32 (19%)	18 (11%)	34 (20%)	21 (13%)	18 (11%)

*N* = 168

*AIC* Akaike information criterion, *BIC* Bayesian information criterion

The chosen model is presented in bold

### Variables associated with the five joint trajectories groups

We examined whether there were differences among the five joint trajectory groups in receptive, expressive and pragmatic language difficulties, all measured at 11 years. This age represents the mid-point of the developmental period examined (7–16 years) and was the first time point at which all three measures of language were available. Gender balance and indicators of parental mental health were also examined. The descriptive statistics and inferential results are summarised in Table 4. Comparisons among the different joint trajectory groups were undertaken. Post hoc group comparisons were carried with a Bonferroni correction applied given these entailed multiple comparisons. In addition, to reduce the number of tests applied, we focused our post hoc comparisons between the problematic trajectory groups (persistent group and increasing peer problems group) and the more favourable trajectory groups (resolving emotional group and low level group). We note that the distribution of data for some of the variables did not meet the assumptions for parametric analyses. Thus, comparisons were repeated using non-parametric statistics. We report robust joint trajectory group differences that were significant after Bonferroni corrections and where the direction of the effect observed remained unchanged when using non-parametric methods.

**Table 4:** Joint trajectory group means (SD) for language, prosociality, gender and parental mental health

	Persistent (*P*) *n* = 44 (26%)	Adolescent onset (AO) *n* = 27 (16%)	Resolving emotional (RE) *n* = 41 (24%)	Increasing peer problems (IPP) *n* = 37 (22%)	Low level (LL) *n* = 19 (11%)	ANOVA/Chi square
Receptive language at age 11	83.82 (15.45)	84.30 (15.08)	84.20 (13.36)	92.23 (15.65)	91.00(18.61)	*F*(4,161) = 2.34
Expressive language at age 11	73.86 (11.53)	76.41 (14.45)	72.15 (9.20)	74.43 (13.18)	71.48(10.09)	*F*(4,161) = 0.74
Pragmatic language at age 11	134.71(13.39)	144.48 (8.86)	143.31 (11.28)	137.89 (13.05)	149.06(8.38)	*F*(4,136) = 6.11^***^
Prosociality at age 11	4.86 (2.49)	6.96 (2.11)	7.54 (2.60)	5.13 (2.20)	7.53 (2.37)	*F*(4, 145) = 9.45^***^
% male	82%	67%	68%	73%	95%	*χ*^2^(4, *N* = 168) = 7.19, *p* = .126
Parental mental health	2.17 (2.71)	0.64 (1.01)	1.00 (1.52)	1.10 (1.61)	0.64 (1.28)	*F*(4,93) = 2.54^*^
% of both parents affected	28%	7%	10%	9%	0%	*χ*^2^(4, *N* = 98) = 8.21, *p* = .084

*p < .05, ***p < .001

No significant differences were found in respect of receptive or expressive language scores. A significant main effect was found for pragmatic language. Post hoc comparisons confirmed that the persistent group had significantly poorer pragmatic language abilities than the resolving emotional and low level groups (persistent vs resolving emotional: *t*(71) = − 8.60, *p* = .004, mean difference − 8.60, (95% CI − 14.41, − 2.80), persistent vs. low level: *t*(52) = − 3.96, *p* < .001, mean difference − 14.35, (95% CI − 21.62, − 7.08). A significant main effect was also found for prosociality. Post hoc comparisons confirmed that the persistent group was significantly less prosocial than the resolving emotional and low level groups (persistent vs. resolving emotional: *t*(74) = − 4.57, *p* < .001, mean difference − 2.67 (95% CI − 3.84, − 1.51); persistent vs. low level: *t*(52) = − 3.71, *p* < .001, mean difference − 2.66 (95% CI − 4.11, − 1.22). The analyses also indicated that the increasing peer problems group had significantly lower prosocial skills than the resolving emotional group and the low levels group [*t*(68) = − 4.11, *p* < .001, mean difference − 2.41 (95% CI,− 3.58 − 1.24) and *t*(46) = − 3.51, *p* = .001, mean difference − 2.40 (95% CI − 3.78, − 1.03), respectively]).

Differences in gender balance among the groups were not significant. Nonetheless, parental reports of their own mental health histories indicated differences between the groups. There was a significant main effect (see Table 4) and post hoc comparisons showed parental reports of their own mental health difficulties were higher for children in the persistent group compared to those in the adolescent-onset group [*t*(41) = 2.03, *p* = .049, mean difference 1.53, (95% CI 0.01, 3.05)], but after Bonferroni correction, this difference was not statistically significant. No other group level comparisons were significant (*p* > .05). Based on visual inspection of the proportions reported by parents of children in the different groups, we also carried out group comparisons on our second measure of parental mental health, i.e., proportion of both parents affected, despite the lack of statistical significance in the overall Chi-square analysis involving all groups [χ^2^ (4, *N* = 98) = 8.21, *p* = .084]. The proportion of both parents affected was higher for children in the persistent group compared to children in the low level group [χ^2^ (1, *N* = 43) = 4.74, *p* = .029]. No other group level comparisons were significant (*p* > .05).

## Discussion

To the best of the authors’ knowledge, this is the first study to examine joint longitudinal trajectories of emotional difficulties and peer relation problems in children with DLD. The findings reveal five distinct patterns of development: (1) low levels of problems in both domains throughout the period studied; (2) childhood onset of problems in both, which remained persistent throughout; (3) adolescent onset in both; (4) low levels of emotional difficulties throughout, alongside increasing peer problems; and (5) emotional difficulties relatively high in childhood and resolving into adolescence, while peer problems were relatively low throughout. This qualifies previous findings based on data aggregated across whole samples [3, 49] and, importantly, reveals that the two areas of difficulty do not invariably occur together.

Slightly over half of the sample did show parallel developments. These were the first three groups listed above. For these children, then, to the extent that there are problems in one of these two aspects of development, there will be problems in the other. This is consistent with the possibility that onset of difficulties in one area promotes difficulties in the other, or with assumptions of bidirectional causality, or with the possibility that a third variable (e.g., underlying common etiological factors, such as genetic factors) explains developments in both areas. These are familiar explanations in developmental psychopathology: it is often the case that children with problems in one area of development have additional problems [50].

The presence of two other groups (together amounting to 46% of the sample), however, complicates the overall picture. In one case, despite relatively high peer problems which increased into adolescence, emotional difficulties were low throughout. For at least some children with DLD, then, peer problems do not precipitate emotional difficulties, and a ‘third variable’ cannot be so straightforwardly attributed responsibility if one domain is seemingly unaffected. Possible interpretations are that these children had sufficiently robust emotional self-regulation or self-efficacy to enable them to withstand emotional problems or that other sources of social support, such as parents, bolstered them against emotional difficulties [16]. In the final group above, peer problems were relatively low throughout, but emotional difficulties were relatively high in childhood and decreased into adolescence. A possible interpretation is that, for these young people, positive peer relations provide a context that, over time, is conducive to the moderation of emotional difficulties [51, 52].

Taken together, these findings lend support to arguments that development in children with DLD is heterogeneous—not only in respect of their language disorder but also in terms of how these are associated with other important aspects of personal and social adjustment. This is important from a theoretical perspective, because it suggests that no one explanation—at least, as currently formulated—can account for all manifestations of DLD and its concomitants [1].

What variables are associated with differing patterns of development of personal and social adjustment in individuals with DLD? We did not find that either comprehension or expressive language difficulties differed among the five joint trajectory groups. It is important to stress that the absence of differences among these groups (all with histories of DLD) does not mean that comprehension or expressive abilities are irrelevant to emotional and peer difficulties [3, 6]. What the present findings do suggest is that, among children with DLD, whatever comprehension or expressive difficulties they have as measured by the instruments used in this study, do not strongly influence which joint trajectory group they fall into.

One aspect of linguistic ability, however, that does appear to be associated with trajectory group membership is pragmatic competence. Children who followed a persistent trajectory, with high levels of emotional and peer problems from childhood to adolescence, had significantly lower pragmatic scores than most of the other groups, and the increasing peer problems group had the second lowest pragmatic scores. More profound limitations in the ability to handle the functional, interpersonal nuances of pragmatic language may put a young person with DLD at a greater risk of following the less favourable joint emotional–peer trajectories. Skills such as making inferences, appropriate conversational turn taking, and tuning into the facial expressions of others are likely to affect emotional recognition [53] and emotional self-regulation [21]. Pragmatic language difficulties are not always apparent to co-locutors, particularly in interaction with peers in childhood. In adolescence, pragmatic difficulties may well be more salient [54]. Adolescents with poor pragmatic skills may thus encounter “demands that exceed capacity” [55]. Adolescents with DLD are likely to experience difficulties processing input from peers about feelings and emotional management, which in turn could lead to feelings of frustration, worry and fearfulness. This argument is further supported by our finding that the children who did not fall into the trajectories defined by peer problem skills (i.e., those in Resolving Emotional and Low Level) and those with peer problems emerging later (i.e., adolescent onset) did not have lower pragmatic competence. It remains for future research to examine whether peer problem-free childhood affords the development of pragmatic skills to a competent level.

We did not obtain clear evidence of a gender imbalance associated with particular trajectory groups. Of particular interest, the findings did not support expectations that proportionally more girls would follow the adolescent-onset trajectory. Population studies report higher levels of depressive symptomatology among teenage girls [25], and we expected that this pattern would be reflected in terms of higher levels of emotional and peer difficulties emerging in adolescence among our female participants. Certainly, many of our participants did show increasing levels of emotional difficulties over time, but this was not a gender-specific outcome. However, it should be acknowledged that, as in most samples of children with developmental language disorder, the proportion of females here was small (24%); future researchers might consider over-recruitment of females to provide more information on the relationship between gender and emotional and peer difficulties in young people with DLD.

The findings with respect to prosociality were also significant. Consistent with expectations, the two least favourable joint trajectory groups (persistent and increasing peer problems in adolescence) did have the lowest mean prosocial scores, and post hoc comparisons between each of these groups and the other joint trajectory groups were statistically significant. Thus, the data not only suggest that lower prosociality accompanies problems in emotional and peer relation domains, but that prosociality is strongly associated with the type of pattern of emotional and peer difficulties that will be followed from childhood to adolescence. We note, however, that these findings are based on the Manchester Language Study (MLS) sample. MLS participants included children with identified developmental language disorders who were receiving support and intervention in language units in childhood. We also note that previous research with the MLS demonstrates that individuals with DLD had continued to develop their expressive and receptive language skills during early adolescence into young adulthood [32]. The early identification of language difficulties coupled with the context of early, intensive language support received in educational contexts such as language units may have nurtured socialisation processes and the development of emphatic concern, which in turn may have influenced the development of prosociality in individuals who participated in the MLS. Indeed, research with the MLS sample suggests that young people with DLD are prosocial and exhibit stable developmental trajectories of prosociality throughout adolescence [56]. It is also important to note, however, that more individual differences in prosociality have been found by other researchers. Lindsay and Dockrell [57], for example, found more individual differences in prosociality in their sample of children with DLD drawn from a variety of schools with different educational provisions in the UK. They found prosocial scores improved between 8 and 12 years of age but worsened by 16 years. Further research with other samples of individuals with DLD, such as community samples or samples of individuals with unidentified DLD would help to unpick the complex relations among these variables over time.

We report preliminary thought-provoking findings that raise the possibility that parental mental health difficulties may be associated with their offspring’s personal and social adjustment. The persistent problems trajectory group had the highest mean score on a measure of parental self-report of their own histories of mental health problems during childhood and adulthood as well as the highest proportion of both parents reporting issues with their mental health. This is consistent with evidence from studies in the general population showing that poorer parental mental health is a predictor of emotional difficulties in children and adolescents [26]. What this paper adds is that, in the context of DLD, this factor may also be associated with concomitant, persistent peer problems. There are a range of potential mechanisms by which parental mental health may be associated with child’s mental health which may be involved in context of DLD. Goodman and Gotlib [59] suggest three mediating and transactional pathways (bio-developmental; psychosocial and contextual) regarding postnatal distress and child emotional and behavioural development which may be worth investigating in future research in this area. It needs to be noted, nonetheless, that in this study we did not have standardised clinical measures of parental mental health with known validity and reliability and the differences observed were preliminary and indicative (see also [58]). Thus, the present finding in this regard should be interpreted with caution. Given the possibility that parental mental health bears on the important aspects of child development in this vulnerable population, the present results warrant further research.

In the same vein, further research could also address some of the limitations present in this study. This investigation used different measures at different ages which may have introduced measurement variability which future research could control for using instruments which span the period of development examined. In addition, minimising attrition so that the same children can be followed across development and maximising completeness of data gathered on associated factors could also be addressed in future longitudinal investigations.

The pattern of findings is important from a clinical perspective. The fact that over half of the sample showed parallel trajectories in emotional and peer domains suggests that diagnosis and monitoring of children with DLD should include examination of much more than language skills. The fact that a large part of the sample showed divergent trajectories across the two domains also warns, however, against assuming that identification of one problem area has clear implications for others; instead, strengths and difficulties need to be identified on an individual basis and potential factors associated with worse outcomes in adolescence. The findings of this investigation also suggest that clinicians should also be sensitive to the possibility that young people experiencing sustained difficulties in both emotional and peer domains may be living in families where there are higher than average levels of parental mental health problems. Furthermore, the difficulties of children with either emotional or peer problems may be less evident than children with both difficulties and professionals need to be vigilant in identifying these needs. In turn, clinical interventions need to take into account the potential breadth of a child’s difficulties, individual areas of robustness/resilience that can be built upon in therapy as well as the potential need for whole family approaches to intervention.

The evidence obtained in this investigation does also offer some positive news concerning emotional and peer difficulties in at least some individuals with DLD. Approximately, 11% of the participants had low levels of difficulties in both domains throughout childhood and adolescence. An additional subset, approximately 24% of the total sample, had emotional problems in childhood that appeared to be resolving during adolescence. These children had low levels of peer problems throughout and also tended to have better pragmatic language scores. Thus, there are encouraging indications not only that some children with DLD do experience relatively favourable trajectories but also that we can identify a particular area of language skills that may be amenable to improvement, with the potential for broader benefits for these young people’s adjustment.

## Electronic supplementary material

Below is the link to the electronic supplementary material.

Supplementary material 1 (DOCX 81 kb)
