# Supplementary material for: Do emotional difficulties and peer problems occur together from childhood to adolescence? The case of children with a history of developmental language disorder (DLD)
Source: Eur Child Adolesc Psychiatry. 2018 Dec 5;28(7):993–1004. doi: 10.1007/s00787-018-1261-6 (PMC6647450; doi:10.1007/s00787-018-1261-6)
Supplement: Supplementary file 1 — Supplementary material 1 (DOCX 81 kb) [file 787_2018_1261_MOESM1_ESM.docx]

**Supplementary Materials Appendix. Specific analyses of trajectories of emotional difficulties**

***Table A1. Model fit statistics and the number and percentages of children assigned to each emotional trajectory group. The 5-group model was the most parsimonious.***

| **Number of groups** | **AIC** | **Sample size corrected AIC** | **BIC** | **Average assignment probability** | **Number (%) of individuals** | | | | | |
| --- | --- | --- | --- | --- | --- | --- | --- | --- | --- | --- |
|  |  |  |  |  | **1** | **2** | **3** | **4** | **5** | **6** |
| 2 | 2888.84 | 2889.54 | 2910.71 | 0.93 | 96 (57%) | 72(43%) |  |  |  |  |
| 3 | 2868.37 | 2869.77 | 2899.61 | 0.85 | 81 (48%) | 40 (24%) | 47 (28%) |  |  |  |
| 4 | 2844.31 | 2846.67 | 2884.92 | 0.80 | 53 (32%) | 52 (31%) | 40 (24%) | 23 (14%) |  |  |
| **5** | **2830.85** | **2834.45** | **2880.85** | **0.80** | **5 (3%)** | **53 (32%)** | **42 (25%)** | **45 (27%)** | **23 (14%)** |  |
| 6 | 2833.86 | 2839.00 | 2893.22 | 0.75 | 5 (3%) | 57 (34%) | 37 (22%) | 28 (17%) | 26 (15%) | 15 (9%) |

Note: *N* = 168

^a^ AIC - Akaike information criterion

^b^ BIC - Bayesian information criterion

***Characteristics of individuals in each of the 5-solution trajectory groups are presented in Table A2. See also Figure A1 below for the graphic representation of the 5-group solution.***

***Table A2*. *Means (SD) and percentages by emotional symptom groups for 5-group solution***

|  | **Very low-level difficulties**  **(n = 23, 14%)** | **Low-level difficulties**  **(n = 45, 27%)** | **Childhood-limited difficulties**  **(n = 42, 25%)** | **Adolescent-onset difficulties**  **(n = 53, 32%)** | **Childhood-onset persistent difficulties**  **(n=5, 3%)** |
| --- | --- | --- | --- | --- | --- |
| Emotional Difficulties |  |  |  |  |  |
| Rutter emotional difficulties age 7 | 0.7 (0.9) | 0.7 (0.9) | 3.6(1.7) | 1.6(1.4) | 5.0(0.7) |
| Rutter emotional difficulties age 8 | 1.4(1.2) | 1.0(1.0) | 3.1(1.7) | 2.6(1.6) | 4.2(2.2) |
| Rutter emotional difficulties age 11 | 0.2(0.4) | 1.4(0.8) | 2.0(1.2) | 3.5(1.3) | 7.4(1.1) |
| SDQ emotional difficulties age 11 | 0.2(0.4) | 1.6(1.0) | 2.4(1.6) | 4.1(1.6) | 8.0(1.2) |
| SDQ emotional difficulties age 16 | 0.2(0.4) | 2.1(1.6) | 1.0(0.9) | 4.7(2.4) | 3.5(1.7) |
| % above borderline cut off for emotional difficulties age 11 | 0% | 0% | 5% | 33% | 100% |
| % above borderline cut off for emotional difficulties age 16 | 0% | 10% | 0% | 48% | 50% |
| Gender, Parental Mental Health,  Language, PIQ and Prosociality |  |  |  |  |  |
| % Male | 87% | 73% | 71% | 75% | 80% |
| Parental Mental Health | 0.5(0.8) | 1.2(1.7) | 0.9(1.6) | 1.7(2.6) | 3.5(2.1) |
| % with both parents affected | 0% | 11% | 9% | 18% | 75% |
| Expressive language age 11 | 74.4(12.1) | 73.2(12.4) | 73.8(10.6) | 74.8(12.4) | 71.0(8.1) |
| Receptive language age 11 | 93.6(19.9) | 89.4(13.7) | 82.5(16.3) | 84.6(13.4) | 86.0(15.9) |
| Pragmatic language age 11 | 146.7(10.9) | 139.8(12.5) | 143.0(11.9) | 139.8(11.4) | 121.4(13.0) |
| PIQ age 11 | 92.2(22.7) | 82.1(22.0) | 88.1(26.4) | 85.2(23.3) | 77.2(16.6) |
| Prosociality age 11 | 6.5(2.5) | 6.3(2.5) | 6.9(2.8) | 5.6(2.6) | 6.6(3.1) |

***Figure A1***. ***Predicted emotional symptom scores on the SDQ scale (5-group solution)***

Note. For ease of interpretation the predicted scores derived for the Rutter have been rescaled to the SDQ scale.
